# The overwhelming role of ballistic photons in ultrasonically guided light through tissue

**DOI:** 10.1038/s41467-022-29157-z

**Published:** 2022-04-06

**Authors:** Eitan Edrei, Giuliano Scarcelli

**Affiliations:** 1 Fischell Department of Bioengineering, University of Maryland, College Park, MD 20742 USA; 2 Department of Applied Physics, The Faculty of Science, The Center for Nanoscience and Nanotechnology, The Hebrew University of Jerusalem, Jerusalem, 91904 Israel

**Keywords:** Microscopy, Optical imaging, Optical techniques, Imaging techniques

**arising from** Maysamreza Chamanzar et al. *Nature Communications* 10.1038/s41467-018-07856-w (2019)

In a recent paper entitled “Ultrasonic sculpting of virtual optical waveguides in tissue”, Chamanzar et al.^1^ presented a method to guide light through scattering media by modifying the refractive index profile within tissue via ultrasound-generated pressure gradients. The authors demonstrated the ability to focus light through 8 mm of a diluted intralipid solution (0.2% intralipid) as well as through 240 µm of mouse cortex. The article claims that while other optical techniques are limited to a depth of hundreds of microns in tissue, their approach can extend confined light delivery to depths of several millimeters. Here we show that the guiding approach described by Chamanzar et al. overwhelmingly affects ballistic photons, and not scattered photons, thus the improvement compared to current optical modalities is marginal at best.

As a striking demonstration of our claim, we show that in similar conditions to Ref. ^1^, one can obtain a confined light spot similar to the one described in the paper by simply focusing light with an external lens. We filled a 1-cm-thick cuvette with a diluted solution of intralipid with a total dilution of 0.2%, i.e., same turbidity conditions described by Chamanzar et al., and focused light (λ = 532 nm) through the sample using an external lens (*f* = 150 mm). The results presented in Fig. 1a show a tightly focal point with a contrast ratio of 5, comparable to that obtained by Chamanzar et al. If external focusing leads to similar light confinement as ultrasonic guiding, then the observed phenomenon is overwhelmingly restricted to ballistic photons, undisturbed by the turbidity of the medium.

**Fig. 1:**
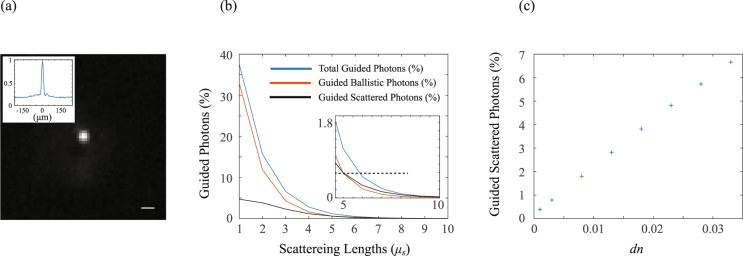
Guided scattered and ballistic photons, experimental and simulated results. **a** A beam focused using an external lens through a 1-cm cuvette filled with a diluted intralipid solution (0.2 %). A sharp focal point with a contrast ratio of 5 is obtained with merely ballistic photons (scale bar = 25 µm). **b** The percentage of guided photons through a waveguide with *dn* = 0.002 and a scattering core of *g* = 0.85. The dashed line in the inset represents a threshold of 0.6%. **c** Percentage of guided scattered photons for increasing values of *dn*.

The fundamental question that needs to be investigated is: under what circumstances is a modification of refractive index profile, by any means, effective to improve light penetration into scattering tissue? To address this issue quantitatively, we performed numerical simulations based on Monte-Carlo ray tracing (*TracePro*). We numerically constructed a waveguide in which the refractive index difference between the core and the cladding is 0.002 similar to the value reported by Chamanzar et al. The core scattering properties (i.e., the scattering coefficient *µ*_*s*_ and the anisotropy parameter *g*) were varied to generate the different scattering conditions. Using this simulation, we quantified the total number of guided rays and identified ballistic vs scattered rays. Note that a certain number of photons will arrive at the output of the waveguide within the core region just by the so-called shower-curtain effect^2^; these photons do not need cladding, are not guided, and should be disregarded. To minimize this effect in our simulation, we used a small radius to length ratio of the waveguide (i.e., *R* = 0.1 mm, *L* = 10 mm), and we confirmed numerically that all photons, either ballistic or scattered, are guided.

The results for *g* = 0.85 (a typical value for tissues such as the mouse cortex^3,4^) are shown in Fig. 1b. To quantify the depth penetration advantage for a given amount of required photons, if we assume that a threshold of 0.6% of the photons is needed to excite a fluorescent label (black dashed line in the inset), guiding only ballistic photons can already excite fluorescence at a depth up to five scattering lengths (red curve); in this scenario, guiding also scattered photons provides a 20% enhancement in depth (blue curve). Our simulation was performed using a step-index rather than the gradient index fiber profile used in Ref. ^1^; however, for a multimode scenario and a similar NA, the difference in index profile is not expected to affect scattering properties but only dispersion^5^, which is immaterial to this discussion, and ray-tracing focusing properties, which mostly rely on ballistic photons.

We then investigated the effect of a larger refractive index difference than the one reported by Chamanzar et al. In Fig. 1c we show simulated results of the percentage of scattered photons guided through five scattering lengths at increasing refractive index differences. We estimate that by inducing a substantial refractive index change (0.03, i.e., more than ten-fold higher than Ref. ^1^) the penetration depth can be increased by twofold. Thus, even under such extreme conditions, the claim that guiding can increase the penetration depth to several millimeters is not justified.

An important additional point: the effectiveness of the method presented by Chamanzar et al. strongly depends on light being scattered predominantly in the forward direction, i.e., high values of *g*. In our simulation, we used the value of *g* = 0.85 that is close to the one reported for the mouse cortex as cited by Chamanzar et al. as well^3^. The high value of *g* also needs to be taken into account when calculating scattering lengths: for instance, the *g* value for the mouse cortex was measured to be 0.86^3^; hence, the number of scattering lengths within 240 µm is 5 and not greater than 7 as reported in Ref. ^1^. Considering the value of *g* and the reduced number of scattering lengths, it is likely that the focal point presented in Figure 7 of the manuscript could have been obtained using external focusing. In various biological tissues, the value of *g* spans a large range and depends on the applied wavelength; in many cases, it averages around 0.9^4,6^, where the simulated increase of depth penetration using the ultrasonic waveguide is expected to be 30%.

In conclusion, light delivery into tissue is not dramatically enhanced by ultrasonic guiding in practical scenarios. We estimate that the guiding demonstrated by Chamanzar et al. provides a marginal enhancement over conventional methods, equivalent to changing the illumination wavelength by approximately 50 nm^7^. The benefit of the ultrasonic guiding, or any guiding based on refractive index modifications within tissue, is limited to samples that scatter light almost solely in the forward direction, and also under these circumstances the addition of guided scattered photons is expected to be minor in terms of total flux arriving to the desired location.

## Data Availability

Data are available upon request.
